# Proteomics of PTI and Two ETI Immune Reactions in Potato Leaves

**DOI:** 10.3390/ijms20194726

**Published:** 2019-09-24

**Authors:** Svante Resjö, Muhammad Awais Zahid, Dharani Dhar Burra, Marit Lenman, Fredrik Levander, Erik Andreasson

**Affiliations:** 1 Department of Plant Protection Biology, Swedish University of Agricultural Sciences, 230 53 Alnarp, Sweden; svante.resjo@slu.se (S.R.); muhammad.awais.zahid@slu.se (M.A.Z.); Dharani.Burra@gmail.com (D.D.B.); marit.lenman@slu.se (M.L.); 2 Department of Immunotechnology, Lund University, 221 00 Lund, Sweden; fredrik.levander@immun.lth.se

**Keywords:** potato, immunity, proteomics, methylation, plants, ETI, PTI, solanum, late blight

## Abstract

Plants have a variety of ways to defend themselves against pathogens. A commonly used model of the plant immune system is divided into a general response triggered by pathogen-associated molecular patterns (PAMPs), and a specific response triggered by effectors. The first type of response is known as PAMP triggered immunity (PTI), and the second is known as effector-triggered immunity (ETI). To obtain better insight into changes of protein abundance in immunity reactions, we performed a comparative proteomic analysis of a PTI and two different ETI models (relating to *Phytophthora infestans*) in potato. Several proteins showed higher abundance in all immune reactions, such as a protein annotated as sterol carrier protein 2 that could be interesting since *Phytophthora* species are sterol auxotrophs. RNA binding proteins also showed altered abundance in the different immune reactions. Furthermore, we identified some PTI-specific changes of protein abundance, such as for example, a glyoxysomal fatty acid beta-oxidation multifunctional protein and a MAR-binding protein. Interestingly, a lysine histone demethylase was decreased in PTI, and that prompted us to also analyze protein methylation in our datasets. The proteins upregulated explicitly in ETI included several catalases. Few proteins were regulated in only one of the ETI interactions. For example, histones were only downregulated in the ETI-Avr2 interaction, and a putative multiprotein bridging factor was only upregulated in the ETI-IpiO interaction. One example of a methylated protein that increased in the ETI interactions was a serine hydroxymethyltransferase.

## 1. Introduction

Plants can be attacked by many organisms, including oomycetes, fungi, and bacteria. They have therefore evolved sophisticated immune systems to protect themselves from invading pathogens, one example of which is the perception of pathogen-associated molecular patterns (PAMPs) via transmembrane pattern-recognition receptors (PRRs). PAMPs have been defined as highly conserved molecules with an important role in the fitness and survival of microbes [1]. Recognition of PAMPs results in activation of PAMP-triggered immunity (PTI), which initiates the induction of immune responses, like the production of reactive oxygen species (ROS), activation of mitogen-activated protein kinase (MAPK) cascades, and transcriptional induction of defense-related genes [2,3]. However, successful pathogens secrete effectors into the plant cell and thereby suppress PTI. This process is called effector-triggered susceptibility (ETS). To counteract this, some plants have also evolved resistance proteins (R proteins) that recognize effectors inside the cell, which results in the initiation of a second level of defense called effector-triggered immunity (ETI) [4]. ETI is associated with a strong immune response, such as programmed cell death at the site of infection, the hypersensitive response (HR), that reduces further spread of the pathogen [5].

PTI and ETI can involve similar defense responses, which include for example activation of kinases and defense gene induction. The difference between PTI and ETI is not clear, and these immune reactions can be considered as a continuum of signaling mechanisms [6]. For example, in a recent study, Leibman-Markus et al. [7] reported a nucleotide-binding leucine-rich repeat receptor (an R like protein), SlNRC4a, to be directly associated with PRRs, which enhances PTI signaling in the absence of effectors.

Although many studies have investigated pathogen-induced changes in transcriptional patterns, there is a lack of information regarding proteomic changes during immune reactions, especially in major crop plants. Since genomic and transcriptomic studies provide no direct information on protein abundance, location, and post-transcriptional alterations, quantitative proteomic analyses are important for our understanding of cellular processes connected to plant defense responses. Protein methylation has only been described in plant immunity with regards to *Arabidopsis thaliana* histone H3, and in this study, a methyltransferase activity was necessary for fully functional immune reactions [8].

Potato late blight is one of the most devastating diseases in potatoes all over the world, and is caused by the oomycete *Phytophthora infestans* [9]. In order to combat late blight disease, there is a need for a detailed understanding of the defense responses to *P. infestans*, including how to combine resistance sources to achieve a sustainable solution. Two studied effectors of *P. infestans*, IpiO and Avr2, both induce HR formation upon recognition by their corresponding plant resistance proteins, the Blb1 and R2 family proteins, respectively [10,11,12]. Avr2 has been shown to associate with a putative phosphatase, BSU-like protein 1 (BSL1), and change brassinosteroid-associated signal transduction [13]. R2 perception of Avr2 is dependent on BSL1 [14]. Both these ETI reactions involve GTP binding and G-protein signaling, as was shown in our earlier proteomics study on one membrane-associated protein fraction [15]. From this proteomics data regarding downstream signaling before the onset of HR, we hypothesize that there are specific changes in protein abundance between different ETIs, similar to the difference between PTI and ETI.

This study aimed to achieve a better understanding of different plant immunity reactions by performing a quantitative proteomics analysis of PTI and two different ETIs (Blb1 and R2) in a protein fraction from a subsequent fractionation of the earlier studied potato immune proteins [15]. We used our established immunity-inducing and protein fractionation systems [15] to quantitatively analyze protein changes and methylation across these different immune reactions.

## 2. Results and Discussion

### 2.1. General Characteristics of the Protein Dataset

**Figure 1:**
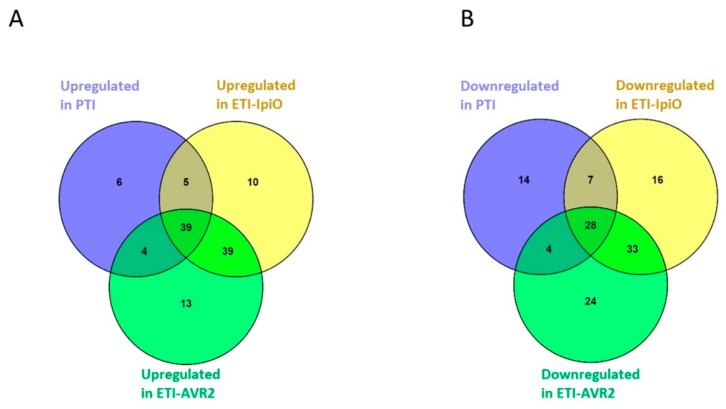
Diagram representing the results of the analysis with the respective number of proteins significantly changed in abundance in PAMP (pathogen-associated molecular patterns) triggered immunity (PTI) and effector-triggered immunity (ETI) interactions (Blb1-IpiO and AVR2-R2), on both (**A**) upregulated and (**B**) downregulated proteins.

In this proteome analysis, 869 proteins were identified and quantified, 243 of which changed in abundance compared to control (*p* < 0.01, supplementary material 1, including GO terms) in at least one of the immune reactions (PTI, ETI-Avir2 or ETI-IpiO). Approximately a third of the quantified proteins displayed the same type of change in all three immune reactions (Figure 1). Another third changed in abundance in both ETI interactions, but not in PTI. The remaining proteins were distributed among the other combinations of conditions, as shown in Figure 1 and Table 1, Table 2, Table 3 and Table 4. In the PTI interaction, we detected an increase in the abundance of 54 proteins, as well as a decrease of 53. As for the ETI response, a total of 39 proteins showed an increased abundance in both ETI-Avr2 and ETI-IpiO interactions (Figure 1). Although protein abundance in the two ETI interactions overlapped, we were able to identify an increase in the abundance of 10 and 13 proteins, and a decrease in abundance of 16 and 24 proteins, that were specific to the ETI-IpiO and ETI-Avr2 respectively (Figure 1). Finally, we also analyzed protein methylation in our dataset, since we found a histone demethylase with decreased abundance in PTI, as well as several histones with decreased abundance in one of the ETI conditions. The overall overlap between the proteins identified in this study and in Burra et al. [15], which describes a different protein fraction of the same biological set up, is limited. In this study, we found 243 proteins that changed in abundance. In the previous study by Burra et al. [15], 183 proteins were found to change in abundance. The overlap between these two sets of proteins is only 22 proteins, indicating that indeed the different fractions contain different proteins.

### 2.2. Proteins with Increased Abundance in the PTI Interaction

Fifty-four proteins were found to be increased in the PTI response (Table 1). Proteins with a sequence similarity to a number of these have previously been shown to be important for PTI or to display an increase in abundance to PAMPs or other elicitors, which indicates that our system is a useful model of potato immune responses.

As one example, we identified a MAR-binding protein (PGSC0003DMP400010835) which was found to be increased in the PTI reaction, specifically. In tomatoes [16], a homologous protein was shown to be induced in response to COS-OGA elicitor treatment. Another protein that increased specifically in PTI and not in ETI interactions was annotated as DUF26 domain-containing protein 1 (PGSC0003DMP400063324). A previous report has shown that a DUF26 domain-containing protein, HvCRK1, is involved in regulating basal resistance but not R-gene dependent programmed cell death in barley [17]. In a recent study, the same protein was found to be increased in abundance when potato plants were challenged with intact *P. infestans* [18].

A metabolic protein that increased was Glyoxysomal fatty acid beta-oxidation multifunctional protein (PGSC0003DMP400008192). In previous studies, it has been shown that in *Arabidopsis*, a mutant lacking glyoxysomal fatty acid beta-oxidation resulted in a reduction of jasmonic acid (JA) accumulation [19], indicating that an increase of this protein might contribute to a generation of signaling molecules needed for the PTI response. Moreover, the identical protein was also found to be increased in abundance when potato leaves were inoculated with *P. infestans* [18]. Another protein that was upregulated in all three interactions was a germin (PGSC0003DMP400031837). Germin-like proteins (GLPs) belong to the functionally diverse cupin superfamily. There is substantial evidence of the involvement of germins and GLPs in general plant defense responses [20,21]. Additionally, transcripts and protein of a close homolog of this germin in *Nicotiana benthamiana* were also found to be increased in abundance after infiltration with disarmed *Agrobacterium tumefaciens* [22].

The protein annotated as UPF0497 (PGSC0003DMP400046973) was also found to increase. UPF0497 is a membrane protein commonly known as CASP (Casparian strip membrane domain proteins)-like protein, which is involved in cell wall strengthening [23]. A previous report has shown that UPF0497 has a higher transcript abundance when Castanea root is challenged with *Phytophthora cinnamomi* [24], which aligns our findings with previous studies and indicates its possible involvement in plant immunity.

Sterol carrier protein 2 (SCP-2) (PGSC0003DMP400014027) showed an increase in abundance, which is interesting since *Phytophthora* species are auxotrophs for sterols, as they lack the ability to produce oxidosqualene [25]. Therefore, it is tempting to speculate that the lower availability of sterols caused by the changed abundance of SCPs is part of immunity.

Moreover, we identified a protease inhibitor-related protein (C6F3B7) and a protease nucellin (PGSC0003DMP400025801) displaying increased abundance. Both protease inhibitors and proteases are known to be involved in PTI responses [26]. Furthermore, two DnaJ chaperone-related proteins (Q2XTC7 and Q6EIX7) were found to be present in higher abundance. The closest homolog to DnaJ-like protein (Q2XTC7) in *N. benthamiana* has earlier shown higher protein abundance upon agroinfiltration [22].

### 2.3. Proteins with Decreased Abundance in the PTI Interaction

Fifty-three proteins were found to be decreased in our proteomics analysis of the PTI interaction (Table 2).

Interestingly, a lysine-specific histone demethylase (PGSC0003DMP400006728) was found to decrease in abundance in the PTI interaction. Lysine demethylases catalyze the demethylation of methylated histones, which in turn can change the degree of DNA condensation and consequently, gene activity [27]. The decreased abundance of histone demethylases in PTI might indicate that increased methylation of histones is a mechanism of immunity and prompted us to further investigate methylation in our dataset.

Two proteins annotated as leucine-rich repeat (LRR) family proteins (PGSC0003DMP400011041, PGSC0003DMP400002269) were found at lower abundance in PTI only. LRR domains are found in many different types of proteins, and these proteins are involved in a variety of biological processes, including R gene immunity [28,29]. LRR domains provide a structural framework for the formation of protein-protein interactions. However, R-proteins generally also contain a nucleotide-binding domain and an NBS domain, and these two potato LRR proteins do not.

The cell wall-related protein xyloglucan endotransglucosylase (Q9FZ05) was found to decrease in PTI and both ETI reactions. β-aminobutyric acid (BABA) is known to induce resistance in potatoes against *P. infestans*, and Q9FZ05 has been shown to decrease when potato leaves are treated with 10 mM BABA [30]. This indicates that decreased levels of specific xyloglucan endotransglucosylases may be part of the defense against pathogens. Other cell wall-related proteins include pectinesterases (PMEs) that catalyze the removal of methyl groups from pectin, leading to loosening of the cell wall. PMEs make pectin more susceptible to microbial enzymes [31], and pectin esterification is correlated with resistance against *Pectobacterium carotovorum* in potato [32,33]. In the present study, pectinesterase (Q9LEB0) was shown to decrease in abundance during all three immune reactions.

Kunitz trypsin inhibitors are known to be involved in plant immunity, and to influence programmed cell death in *A. thaliana* [34]. In our study, a protein annotated as Kunitz-type protease inhibitor (PGSC0003DMP400016825) decreased in abundance in the PTI and ETI-Avr2 interactions. In a previous RNA study, a close homolog of this Kunitz-type protease inhibitor in *N. benthamiana* was also decreased in abundance upon infiltration with *A. tumefaciens* [22]. We also identified a protease, xylem serine proteinase 1 (PGSC0003DMP400012806) that decreased in abundance in PTI and both ETIs. We performed quantitative RT-PCR with primers directed to xylem serine proteinase 1 as a validation experiment for expression of this gene and confirmed that the transcript also decreased in abundance. The average fold change in expression in the PTI and ETI plants, as compared to control plants infiltrated with media, was 0.27 for PTI, 0.18 for ETI-IpiO, and 0.16 for ETI-Avr2, *p* = 0.015. Furthermore, xylem serine proteinase 1 was found to decrease in abundance in a recent study by Xiao et al. [18], where they inoculated potatoes with *P. infestans.* A possible explanation of the decrease in abundance of Kunitz-type protease inhibitor and xylem serine proteinase 1 could be that they are part of fine-tuning the immunity or HR processes, which require some negative regulators [35].

### 2.4. Proteins with Increased Abundance in ETI Interactions

Thirty-nine proteins increased in abundance in response to both ETI-Avr2 and ETI-IpiO interactions, but not in PTI (Figure 1, Table 3). Despite the significant overlap between proteins regulated by the two ETI interactions, we have identified 10 and 13 proteins that specifically increased in ETI-IpiO and ETI-Avr2, respectively (Figure 1). Among them, a putative multiprotein bridging factor (A0MWB6), with a DNA binding domain, was explicitly increased in the ETI-IpiO interaction. Similar proteins have been hypothesized to link ROS signaling, lipid metabolism, and pathogen defense [36]. In line with this, a family of catalase proteins (PGSC0003DMP400002845, Q6RFS8, and Q2PYW5) were found to increase in the ETI interactions. Catalase (CAT) is an iron porphyrin enzyme, which serves as a scavenger of reactive oxygen species (ROS) [37], and CAT related genes have been found to be regulated by biotic and abiotic stresses [38]. For example, the closest homolog in sugarcane, ScCAT2, was found to play a positive role in immune responses during plant–pathogen interactions [39]. The increase of CAT proteins, together with the putative multiprotein bridging factor, makes it tempting to speculate that ROS tolerance mechanisms are activated during ETI interaction. A protein involved in lipid transport, P-rich protein EIG-I30 (PGSC0003DMP400024366), was found to have increased abundance in ETI interactions. Previously, a closely related protein from the same family was shown to increase after BABA treatment of potato leaves [30]. The increase of P-rich protein EIG-I30 in the present study suggests its association with the defense-related response in ETI interactions of potato.

Our analysis of the proteins specifically increased in ETI interactions revealed the presence of proteins with RNA binding activity, such as mRNA binding protein (PGSC0003DMP400022826), heterogeneous nuclear ribonucleoprotein 27C (PGSC0003DMP400029964), and an ATP-dependent RNA helicase 8 (Q8RXK6). There is increasing evidence demonstrating a role for RNA-related proteins in regulating plant immunity [40]. Relating to the ATP-dependent RNA helicase 8 (Q8RXK6) that was specifically increased in ETI-Avr2, overexpression of the closest rice homolog OsBIRH1 in *Arabidopsis* resulted in plants with enhanced resistance against *Alternaria brassicicola* and *Pseudomonas syringae* pv. tomato DC3000 [41].

Another protein with increased abundance was carbonic anhydrase (Q5NE20). In a previous study, silencing of its closest homolog in *N. benthamiana* increased susceptibility to *P. infestans* [42]. Finally, heat shock protein 70 (PGSC0003DMP400015694) was found to increase in the ETI-Avr2 interaction. This type of protein has also been reported to interact with R-gene encoded proteins [43], which is in accordance with its increased abundance in the ETI interaction. Knowledge about these kinds of associations in major host plants such as potato is important since for example HSP70 is a large gene family and it is, therefore, challenging to know the exact target in new breeding and biotechnology.

### 2.5. Proteins with Decreased Abundance in the ETI Interaction

Seventy-three proteins decreased in at least one of the ETI conditions, but not in PTI (Table 4). An endoglucanase (Q42871) displayed decreased abundance in both ETI interactions. Its closest homolog in tomatoes, Cel1, has been found to decrease upon fungal infection, indicating a possible role in plant-pathogen interactions in solanum [44]. In another study, Flors et al. [45] found that when the endo-beta-1,4-glucanases Cel1 and Cel2 were lacking, susceptibility to Botrytis cinerea in tomatoes was decreased. Both of these findings indicate that decreased abundance of the Q42871 endoglucanase can be part of a successful immune response in potatoes.

The metabolic enzyme ferredoxin-dependent glutamate synthase 1 (PGSC0003DMP400017124) displayed decreased abundance in ETI. As a validation experiment for this gene, quantitative RT-PCR with primers directed to ferredoxin-dependent glutamate synthase 1 also revealed decreased transcript levels. The average fold change of expression in the PTI and ETI plants, as compared to control plants infiltrated with media, was 0.42 for PTI, 0.35 for ETI-IpiO, and 0.07 for ETI-Avr2, *p* = 0.009. It is likely that this decrease reflects a changed need for active amino acid metabolism during immunity [46].

We found some proteases, subtilases, and PR proteins to be decreased in ETI reactions. They belonged to protein families that have members that previously have been reported to increase in different types of immunity. Two possible explanations are that these specific family member proteins may have translocated to a different subcellular compartment, where they will be active during the immune response, or that these family members have a negative role. It is known that the subtilisin type protease AtSBT5.2 gene can produce two alternative proteins, a secreted protease AtSBT5.2 (a) and an intracellular AtSBT5.2 (b) protein. This latter form of the protein interferes with a defense gene-inducing transcription factor, leading to suppression of HR and impaired resistance [47].

A protein annotated as aspartic proteinase nepenthesin-1 (PGSC0003DMP400032897) displayed decreased abundance during the ETI-IpiO interaction only. In another proteomics study, its closest homolog in black pepper also showed decreased abundance when challenged with *Phytophthora capsici* [48]. These findings suggest that decreased abundance of an aspartic proteinase is possibly involved in plant defense mechanisms. Furthermore, we have identified a Lysyl-tRNA synthetase (PGSC0003DMP400046710) and a eukaryotic translation initiation factor (PGSC0003DMP400013944) which decreased in abundance during the ETI-IpiO condition.

In the ETI-Avr2 interaction, 24 proteins displayed decreased abundance specifically. Among them, four histones decreased: histone H1, histone H2A.1, histone H2A, and histone H1F. It is interesting that all histones identified in this study display a distinct change in abundance in only one type of immune response. One possible explanation is that these histones may have undergone post-translational modifications that have changed their affinity for chromatin, making them less likely to be isolated by our sample preparation procedure. Histones undergo a large number of post-translational modifications, such as methylation and acetylation. These modifications can affect the degree of chromatin relaxation, and thus may change how histones bind to chromatin.

### 2.6. Protein Methylation

Protein methylation on lysine and arginine residues is a tightly controlled process that contributes to the regulation of protein function in several different ways. Lysine and arginine methylation is catalyzed by methyltransferases and demethylases. One of the most well-characterized biological roles of protein methylation is the effect of methylation of histones. This modification can regulate gene activity, either positively or negatively. We observed both a reduction of lysine-specific histone demethylase in PTI and a specific decrease in abundance of a number of histones in ETI-Avr2. Methylation is known to affect binding to chromatin. Due to this, we decided to investigate protein methylation in our dataset. We identified 40 high confidence methylated peptides from 34 methylated proteins in our samples (Table 5, supplementary material 1). For example, we identified one histone methylation, on Lys 116 of histone H3 (H3116me). This lysine residue is located in the protein core of the histone, and not in the n-terminal part where most well-characterized methylations are found [27]. We have not been able to find any publication describing methylation of histone H3 on this site, making this the first observation of this particular methylation site. Notable among the other methylated proteins were histone acetyltransferase, which catalyzes the acylation of histones. This is an important step in chromatin relaxation, leading to increased gene transcription, as well as a number of chloroplast proteins such as rubisco large chain and chlorophyll a-b binding protein, which have previously been shown to be methylated in *Arabidopsis* [49].

We identified other methylated proteins that changed in abundance during immune reactions, including UPF0497 membrane protein (CASP-protein) (PGSC0003DMP400046973) and serine hydroxymethyltransferase (P50433), which both increased in the ETI conditions. The UPF0497 membrane protein has been discussed earlier, and it is possible that the activity of this protein is regulated by methylation, even though we have not found any report of this in the literature. Metabolism and methylation status are linked in several ways in plants [50]. The serine hydroxymethyltransferase catalyzes the methylation of tetrahydrofolate in the folate cycle. This methyl group can be transferred into the methionine cycle, where it is used to produce S-adenosylmethionine (SAM), which is the main methyl donor in protein and DNA methylation reactions, linking serine hydroxymethyltransferase to methylation reactions [51]. The inhibition of the folate cycle has been demonstrated to cause reduced DNA and histone methylation in *Arabidopsis* [52]. Methionine synthetic pathways have also been shown to be upregulated during the plant immune response [53].

## 3. Materials and Methods

### 3.1. Plants and Infiltration

The methodology used in this study has been thoroughly described in our previous study by Burra et al. [15]. Three different types of *Solanum tuberosum* cv. Désirée were used in this study, namely the wild type Désirée plants, AO1-22 (Désirée with the Rpi-Blb1 resistance gene) and T16 (Désirée with the R2-type resistance gene) [54,55,56]. Plants were grown in vitro for 2 weeks (MS media with vitamins, 16 h of light, 23 °C day temperature, 18 °C night temperature) followed by 4 weeks in soil (22 °C, 16 h of light), as described in Abreha et al. [54] and Burra et al. [15]. The Agrobacterium AGL1 strains were grown on 10 mL YEB medium supplemented with 1 µL of 200 mM acetosyringone, 100 µL of 1 M MES buffer and antibiotics for 24 h at 28 °C, 200 rpm until an OD600 of 1, as explained in Du et al. [57] and Burra et al. [15]. After this, the bacteria was re-suspended in infiltration media (5 g/L MS salts, 1.95 g/L MES, 20 g/L sucrose, 200 µM acetosyringone, pH 5.6). Infiltration with infiltration media occurred only for the control or bacterial suspension with OD600 = 0.3 and was done on the abaxial surface of the leaflets of four plants per genotype, as described by Burra et al. [15]. The Agrobacterium transformed with either an empty vector into wild type plants (the PTI condition), the IpiO effector gene into AO1-22 plants (the IpiO-Blb1 condition), or the Avr2 effector gene into T16 (the Avr2-R2 condition). The macroscopic cell death phenotype was assessed at 18, 42, and 72 h post infiltration (hpi), and the whole experiment was repeated twice. The plants showed no visible symptoms at 18 hpi, while an even cell death was found in both ETI interactions at 42 and 72 hpi (data not shown, Burra et al. [15]). This study has followed local, national, and international guidelines and legislations, and all required or proper permissions and/or licenses were obtained.

### 3.2. Protein Fractionation

Eight biological replicates originating from two independent experiments were processed. Each sample consisted of two stabs from two leaflets 18 h post infiltration (hpi), corresponding to 100 mg fresh weight. Each sample was cooled on ice and put in a 1.5 mL Eppendorf tube with sea sand, before processing with a Subcellular Protein Fractionation Kit for Tissues (ThermoFisher Scientific, Waltham, MA, USA, Catalog No. 87790) with minor modifications (see below, and Burra et al. [15]). Briefly, proteins were consecutively extracted in four different buffers included in the kit, and the final supernatants were frozen at −80 °C until further use. Each leaf sample was disrupted using pestle sticks in 1 mL ice-cold cellular extraction buffer (CEB). The sample was then passed through tissue and centrifuged at 500× *g* for 5 min at 4 °C. The 500× *g* CEB pellet was washed and centrifuged once with CEB, and ice-cold membrane extraction buffer (MEB) was added to the washed pellet. The pellet was then vortexed and incubated at 4 °C for 10 min with gentle mixing. After incubation, the solution was centrifuged at 3000× *g* for 5 min. The supernatant was cleared by re-centrifugation at 16,000× *g* for 10 min at 4 °C and the supernatant saved as the membrane fraction, which is the fraction that was analyzed in Burra et al. [15]. The pellet obtained after the 3000× *g* centrifugation was washed once with MEB and centrifuged. To the resulting pellet, ice-cold nuclear extraction buffer (NEB) was added, and the sample was vortexed and incubated for 30 min at 4 °C with gentle mixing. After incubation the supernatant was cleared by re-centrifugation at 16,000× *g* for 10 min at 4 °C and the supernatant was analyzed in this paper.

### 3.3. Tryptic Digestion and Mass Spectrometry

Proteins were separated on a 14% SDS-PAGE gel. The whole lane was removed and washed, and the proteins were digested using trypsin (Promega Trypsin Gold, Madison, WI, USA, Mass Spectrometry Grade Trypsin Gold, Catalog number: V5280). The digests were desalted by C18-based spin columns (The Nest Group Inc., Southborough, MA, USA) as described in Chawade et al. (2016), and analyzed with the application of an Eksigent nanoLC2D HPLC system with an online LTQ Orbitrap XL ETD [58]. The full procedure is detailed in Burra et al. [15].

### 3.4. Peptide Data Analysis

The raw data from the Orbitrap was converted to Mascot generic files (mgf) with ProteoWizard [59]. A protein database consisting of solanum proteins from UniProt (www.uniprot.org), downloaded 24 August 2011, protein sequences from the Potato Genome Project [60] and the Agrobacterium proteins from UniProt, downloaded 10 March 2015, concatenated with an equal size decoy database (random protein sequences with conserved protein length and amino acid distribution, in total 36,512 target and decoy protein entries), was generated using a modified version of the decoy.pl script from MatrixScience (http://www.matrixscience.com/help/decoy_help.html) [61]. The mgf files were used for searches against this database with Mascot version 2.3.01 in the Proteios software environment [62]. Search tolerances were 7 ppm for precursors and 0.5 Da for MS/MS fragments. For the searches used for quantitative analyses, one missed cleavage was allowed, and carbamidomethylation of cysteine residues was used as fixed modification and oxidation of methionines as variable modification. For the identification of methylated proteins, carbamidomethylation of cysteine was used as a fixed modification, while oxidation of methionines, mono-, di-, and trimethylation of lysine and mono-, di-, and trimethylation of arginine were used as variable modifications. Search results were exported from Mascot as XML, including query level results, with a modification to the export script to include protein accession numbers, and also for the query (spectrum) level results. The results were imported to Proteios, where q values were calculated using the target-decoy method described by Käll et al. [63]. The search results were then filtered at a peptide-spectrum match q-value of 0.01 to obtain a false discovery rate of 1% in the filtered list. For quantitative peptide analysis, a label-free approach based on precursor ion intensities was used [64], with all data processing steps performed within Proteios. MS1 peptide feature detection was performed using Dinosaur [65], while the other data processing steps were performed in Proteios, and subsequent feature matching and alignment between LC-MS/MS was run with a previously described workflow [66]. The resulting peptide data were normalized using Loess-G normalization [67] in the Normalyzer software [68]. The normalized data were analyzed using DanteR [69]. For methylated peptides, a separate FDR calculation was performed. The matches against methylated peptides were sorted by order of decreasing Mascot score, and for each peptide the total number of peptides with an equal or higher score was calculated. The methylFDR for a given hit was then calculated by dividing the number of decoy hits by the total number of hits. To produce a final list of methylated peptides, the hits were then filtered at a methylFDR value of <0.01. To increase the confidence of our final list, we excluded tri- and dimethylated lysines. Trimethylated lysine was excluded since it is isobaric with acetylated lysine. Dimethylated lysine is isobaric with arginine and was excluded since there is a risk that a spectrum from the fragmentation of an arginine-containing peptide may be misidentified as that of a peptide containing dimethylated lysine, if such a peptide is present in a homologous protein also included in the database. We also required that all peptides should be the top hit for their respective spectrum, to decrease the risk of misidentification due to sequence differences that are isobaric with methylation (i.e. I/L to V). In order to be able to differentiate between peptides where the methylation site was determined and peptides that were methylated but the methylation site could not be identified, we adopted the Mascot delta score method [70], with a DS > 10 regarded as an identified methylation site. The data were deposited in the PRIDE database (PXD012576).

### 3.5. RNA Extraction and Quantitative RT-PCR

RNA from potato leaf tissue samples was isolated using the Qiagen RNeasy Plant Mini kit, according to the manufacturer’s protocol. RNA was quantified using Nanodrop Spectrophotometry (Table S1, Figure S2), and 500 ng RNA was used for cDNA synthesis using SuperScript III (Thermofisher, Waltham, MA, USA, Catalog No. 18080051) with oligo dT primers. cDNAs were diluted 10 times with nuclease-free water prior to qPCR analysis in a CFX96 real-time thermal cycler (Bio-Rad). qPCR was performed on four biological and three technical replicates using Platinum SYBR Green qPCR SuperMix kit (Thermofisher, Waltham, MA, USA, Catalog No. 11733038). All qPCR data were analyzed using the Pfaffl-method [71], with expression normalized to the housekeeping gene Ef1α and fold change calculated using primer-pair amplification efficiencies determined from standard curves. A one-way ANOVA and F-test were used to analyze the data. Primers were designed using Primer3 [72], with sequence information from http://solanaceae.plantbiology.msu.edu. The primers were the following: for the housekeeping gene Ef1α_F (5’ GAACTGTCCCTGTTGGTCGT 3’) and Ef1α_R (5’ GGGTCATCCTTGGAGTTTGA 3’), for ferredoxin-dependent glutamate synthase 1, P17124_F (5’ GGATTGGTTATGCGGCAACT 3’) and P17124_R (5’ TTTGCGATAAAACCGACCC 3’), and for xylem serine proteinase 1, P12806_F (5’ TCCCCTCTTGGCTTCATGT 3’), P12806_R (5’ GCTTGATGAGGGGTGAGAA 3’). Primer efficiencies, agarose gel analysis of PCR products, and generated melting curves are shown in Figure S2.

## 4. Conclusions

In this paper, we described an analysis of one PTI and two ETI models in potatoes and compared their resulting changes in protein abundance. In PTI, germin, proteases, and a CASP-like protein increased, all in line with what is expected during the initial stages of immunity. Sterol carrier protein 2 increased in abundance in all three immune reactions, which is interesting since *P. infestans* and other oomycete pathogens rely on the host for sterols. Several proteins with RNA binding activity had higher abundance in ETI, in line with increasing evidence demonstrating a role for RNA-related proteins in regulating plant immunity. In the ETI interactions, several catalase proteins were found to be higher in abundance, which can perhaps be linked to ROS tolerance mechanisms. We showed that four histones decrease in abundance, specifically in the ETI-Avr2 interaction, and that no histones display changes in abundance in the other immune reactions. We also demonstrated the first proteomic analysis of protein methylation in potatoes, including a previously unidentified methylation site on histone H3 and the methylation of serine hydroxymethyltransferase, which increases in the ETI interaction. The serine hydroxymethyltransferase participates in the pathway producing the main methyl donor in methylation reactions.

Data from our earlier proteomics study on one membrane-associated protein fraction [15] indicated that specific protein differences between different ETIs are in a similar range to the differences between PTI and ETI at downstream signaling, before the onset of HR. The current data support the idea that downstream signaling differs between different ETIs to a similar degree as ETI differs from PTI. Our methods and results could be used in future mechanistic-based pre-breeding and prediction of sustainable combinations of resistance genes by, for example, potato single reaction monitoring SRM [58]. Resistance genes are known to be problematic with regards to durability; the pathogen can overcome this type of resistance. Therefore, efforts are being made to combine different sources of resistance [73]. Criteria for selection of combinations of resistance sources are the host range and how broad it is, and a future selection criterion can be using genes or genetic material with different resistance mechanisms. This study lays the foundation for the last criterion and could be used even if the gene is unknown by combining different down-stream mechanisms.

## Figures and Tables

**Table 1:** Proteins with increased abundance from PTI and two ETI interactions, 18 hpi (hours post infiltration). A comparison was made between the immune-treated plants and the control plants infiltrated with medium only. All values are in log2 fold. Non-significant *p* values are indicated by NA. All *p*-values < 0.01 are shown in the table.

Protein ID	Protein Name	Degree of Regulation (log2)		
		PTI	ETI (Blb1-IpiO)	ETI (AVR2-R2)
PGSC0003DMP400050772	Conserved gene of unknown function	4.57	5.07	4.63
P00296	Plastocyanin	4.47	5.10	4.88
Q8RXR5	Polyadenylate-binding protein 2	3.20	3.79	4.20
PGSC0003DMP400046973	UPF0497 membrane protein	3.17	3.13	3.41
Q2XTE0	Chlorophyll a-b binding protein	3.07	4.51	4.81
PGSC0003DMP400047776	Protein translocase secy subunit	2.97	2.80	3.01
PGSC0003DMP400022300	Chlorophyll binding protein CP24 10B	2.76	3.41	3.05
Q2XTC7	DnaJ-like protein-like	2.75	2.94	3.13
P27489	Chlorophyll a-b binding protein 13	2.57	4.21	4.37
PGSC0003DMP400011243	Sodium/ exchanger	2.56	2.85	2.65
Q2MI72	Photosystem II reaction center protein	2.45	3.30	3.18
Q0PWS7	Chlorophyll a-b binding protein	2.22	3.51	3.67
P10708	Chlorophyll a-b binding protein 7	2.17	3.28	3.32
C6F3B7	Protease inhibitor-related protein	2.08	2.46	1.83
P32764	Rubisco small chain 3	2.04	2.16	2.33
P54260	Aminomethyltransferase	1.93	1.82	1.96
Q94KR9	Translation initiation factor IF-1	1.77	1.77	1.86
Q9FFG6	AT5g05480/MOP10_2	1.75	1.10	1.24
Q2MI49	Photosystem I iron-sulfur center	1.75	2.26	1.94
D2K7Z2	Photosystem I reaction center subunit	1.75	2.26	2.11
Q7YJ37	Cytochrome b559 subunit alpha	1.71	2.67	2.38
Q2MI71	Cytochrome b6	1.67	1.93	1.85
PGSC0003DMP400017273	Brg-1 associated factor	1.66	2.11	2.25
E8ZG61	Pectinesterase	1.62	1.22	1.49
PGSC0003DMP400031837	Germin	1.57	1.43	1.36
PGSC0003DMP400033355	Mitochondrial import inner membrane translocase subunit Tim9	1.52	1.48	1.35
Q0PWS5	Chlorophyll a-b binding protein	1.48	1.80	1.52
PGSC0003DMP400030419	Heteronuclear ribonucleoprotein A1	1.44	2.11	2.19
P12360	Chlorophyll a-b binding protein 6A	1.43	1.76	1.58
PGSC0003DMP400034002	Rubisco small chain 1	1.40	1.73	1.73
Q9SL05	Protein proton gradient regulation 5	1.36	1.69	1.31
P93014	30S ribosomal protein S5	1.33	1.62	1.38
Q2VEI0	Photosystem II CP43 protein	1.29	1.74	1.82
Q2MI75	Photosystem II CP47 protein	1.24	1.80	1.93
PGSC0003DMP400053532	116 kD U5 small nuclear	1.15	1.24	1.48
PGSC0003DMP400038531	Chloroplast photosystem I, protein V	1.04	1.95	1.88
PGSC0003DMP400014027	Sterol carrier protein 2	0.99	1.19	1.54
Q2UVD9	PSII cytochrome b559 8kDa subunit	0.89	1.51	1.58
Q2VEH0	ATP synthase subunit beta	0.51	0.88	1.10
Q6EIX7	Potyviral capsid interacting protein 2b	2.14	1.57	NA
B3F8I0	Glyceraldehyde-3-phosphate dehydrogenase	1.56	1.48	NA
PGSC0003DMP400043522	Peroxidase 12	1.37	1.02	NA
PGSC0003DMP400048828	Splicing factor	1.29	1.25	NA
PGSC0003DMP400039723	(S)-2-hydroxy-acid oxidase	1.14	0.96	NA
P93363	Tyrosyl-tRNA synthetase	1.73	NA	1.44
PGSC0003DMP400025801	Nucellin	1.48	NA	1.48
PGSC0003DMP400044750	CXE carboxylesterase	1.07	NA	0.87
Q0WVD8	Adenylate translocator	0.63	NA	0.52
E1AXT8	Glycolate oxidase	1.71	NA	NA
PGSC0003DMP400000661	Beta-galactosidase	1.42	NA	NA
PGSC0003DMP400010835	MAR-binding protein	1.35	NA	NA
PGSC0003DMP400063324	DUF26 domain-containing protein 1	1.33	NA	NA
Q8LK04	Glyceraldehyde-3-phosphate dehydrogenase	1.02	NA	NA
PGSC0003DMP400008192	Glyoxysomal fatty acid beta-oxidation multifunctional protein	0.75	NA	NA

**Table 2:** Proteins with decreased abundance from PTI and ETI interactions, 18 hpi (hours post infiltration). A comparison was made between the immune-treated plants and the control plants infiltrated with medium only. All values are in log2 fold. Non-significant *p* values are indicated by NA. All *p*-values < 0.01 are shown in the table.

Protein ID	Protein Name	Degree of Regulation (log2)		
		PTI	ETI (Blb1-IpiO)	ETI (AVR2-R2)
Q38JJ2	Disulfide-isomerase-like protein	−0.59	−1.02	−0.71
Q5M9V4	ATP synthase subunit alpha	−0.60	−0.87	−0.87
Q0WN54	Molecular chaperone Hsp40/DnaJ	−0.64	−0.82	−0.79
Q9ZR78	ATP synthase subunit beta	−0.66	−0.91	−0.87
PGSC0003DMP400042639	Conserved gene of unknown function	−0.72	−0.96	−0.86
PGSC0003DMP400050963	Chlorophyll a oxygenase	−0.76	−0.77	−0.78
PGSC0003DMP400053725	Ribonucleoprotein. chloroplast	−0.78	−1.05	−0.77
PGSC0003DMP400053197	ATP synthase subunit beta	−0.82	−0.83	−0.97
PGSC0003DMP400005664	Elongation factor Ts	−0.84	−1.61	−1.24
PGSC0003DMP400010972	Hydroxypyruvate reductase	−0.84	−1.03	−0.74
PGSC0003DMP400054045	Alpha-l-fucosidase	−0.87	−0.90	−0.80
PGSC0003DMP400002234	30S ribosomal protein S1	−0.89	−0.94	−0.74
PGSC0003DMP400008070	Ubiquitin-associated	−0.93	−0.84	−0.76
PGSC0003DMP400001305	Protein disulfide isomerase family	−0.98	−1.29	−0.86
Q2XPV6	Phosphoglycerate kinase	−1.04	−0.94	−0.75
A8MQR4	60S acidic ribosomal protein P0	−1.05	−1.10	−0.74
Q9LEB0	Pectinesterase	−1.05	−1.04	−1.08
Q40460	Ribulose bisphosphate carboxylase	−1.05	−1.39	−1.41
PGSC0003DMP400031911	Pre-mRNA splicing factor	−1.06	−1.08	−0.89
C0SUW8	Eukaryotic translation initiation factor	−1.07	−0.77	−0.92
PGSC0003DMP400027836	Alpha-glucosidase	−1.08	−1.28	−1.33
PGSC0003DMP400014012	Annexin	−1.19	−1.25	−1.21
PGSC0003DMP400012806	Xylem serine proteinase 1	−1.21	−1.02	−1.08
PGSC0003DMP400054512	Cell division inhibitor	−1.29	−1.00	−0.95
PGSC0003DMP400019687	Translation initiation factor IF-3	−1.37	−1.42	−0.94
PGSC0003DMP400038185	Transketolase	−1.52	−1.10	−0.77
Q9FZ05	Xyloglucan endotransglucosylase	−1.71	−0.86	−1.18
Q84V30	Phosphatidylserine decarboxylase proenzyme 1	−2.00	−1.58	−1.80
O81394	Phosphoglycerate kinase	−0.62	−0.59	NA
Q6J995	Chloroplast glutamine synthetase	−0.96	−0.61	NA
PGSC0003DMP400001471	Multicopper oxidase	−1.00	−1.03	NA
F4JNJ2	NAD(P)-binding protein	−1.05	−0.72	NA
B0FPD8	Fructose-bisphosphate aldolase	−1.16	−0.89	NA
P93565	Fructose-bisphosphate aldolase	−1.17	−0.98	NA
Q5GM68	Phosphoenolpyruvate carboxylase 2	−1.33	−0.94	NA
F4J912	Ribosomal protein L5	−0.93	NA	−0.73
Q41499	Spliceosomal protein	−1.03	NA	−0.53
PGSC0003DMP400048033	Heme-binding protein	−1.14	NA	−0.90
PGSC0003DMP400016825	Kunitz-type protease inhibitor	−1.21	NA	−1.20
PGSC0003DMP400002269	Leucine-rich repeat protein	−0.62	NA	NA
Q2MIB4	ATP synthase subunit b	−0.64	NA	NA
Q9SKI2	Vacuolar protein	−0.69	NA	NA
PGSC0003DMP400052035	KH domain-containing protein	−0.72	NA	NA
PGSC0003DMP400032499	Multicopper oxidase	−0.80	NA	NA
PGSC0003DMP400029544	Serine-threonine protein kinase	−0.82	NA	NA
PGSC0003DMP400037531	Small nuclear ribonucleoprotein	−0.87	NA	NA
Q38M62	Uncharacterized protein	−0.87	NA	NA
PGSC0003DMP400036013	Nucleic acid binding protein	−1.01	NA	NA
PGSC0003DMP400020414	Glyceraldehyde-3-phosphate de	−1.13	NA	NA
PGSC0003DMP400011041	Leucine-rich repeat family protein	−1.23	NA	NA
C0Z2D8	AT1G20440 protein	−1.38	NA	NA
PGSC0003DMP400004729	NMDA protein	−1.68	NA	NA
PGSC0003DMP400006728	Lysine-specific histone demethylase	−2.00	NA	NA

**Table 3:** Proteins from two ETI interactions with increased abundance (ETI-IpiO and ETI-Avr2), 18 hpi (hours post infiltration). A comparison was made between the immune-treated plants and the control plants infiltrated with medium only. All values are in log2 fold. Non-significant *p* values are indicated by NA. All *p*-values < 0.01 are shown in the table.

Protein ID	Protein Name	Degree of Regulation (log2)	
		ETI (Blb1-IpiO)	ETI (AVR2-R2)
PGSC0003DMP400024366	P-rich protein EIG-I30	4.45	5.3
PGSC0003DMP400015464	Chlorophyll a/b binding protein	6.24	5.03
PGSC0003DMP400007091	Acetylglutamate kinase	2.8	3.76
Q40430	PSI-H	3.07	3.35
Q7M1K8	Chlorophyll a-b binding protein	3.01	3.18
PGSC0003DMP400029632	ATP synthase delta chain	2.49	2.69
PGSC0003DMP400002845	Catalase isozyme 2	1.95	2.21
P06183	Photosystem II 10 kDa polypeptide	2.16	2.2
Q9SR73	40S ribosomal protein S28-1	2.14	2.16
P14278	Chlorophyll a-b binding protein 4	2.06	2.14
Q9S7N7	PS I reaction center subunit V	2.19	2.12
Q3S492	Proteinase inhibitor I	1.97	1.8
Q2MIA5	Photosystem II D2 protein	1.75	1.78
PGSC0003DMP400022826	MRNA binding protein	1.62	1.54
PGSC0003DMP400013603	Translation initiation factor IF-1	1.25	1.51
Q70PN9	Putative PSI-D subunit	1.49	1.42
PGSC0003DMP400034978	PS I reaction center subunit IV isoform	1.36	1.34
B8XLF1	Chlorophyll a-b binding protein	1.82	1.32
PGSC0003DMP400030353	Cytosolic acetoacetyl-coenzyme A thiolase	1.26	1.28
F1KC21	Photosystem II protein D1	1.16	1.23
PGSC0003DMP400012170	ATP synthase subunit O	1.16	1.21
PGSC0003DMP400030249	Chloroplast protease	0.99	1.2
PGSC0003DMP400048099	Glycolate oxidase	1.03	1.19
PGSC0003DMP400031997	Photosystem II 11 kDa protein	1.04	1.19
P25079	Ribulose bisphosphate carboxylase large chain	0.95	1.13
PGSC0003DMP400030421	Heterogeneous nuclear ribonucleoprotein A1	1.37	1.11
PGSC0003DMP400025599	Conserved gene of unknown function	0.95	1.05
Q2MIA8	DNA-directed RNA polymerase beta	0.99	0.98
Q69GY7	Cytochrome b6-f iron-sulfur subunit	0.97	0.92
PGSC0003DMP400052418	Gamma-glutamyl transferase	1.15	0.9
PGSC0003DMP400017746	Oxygen-evolving enhancer protein 1	0.97	0.89
Q9S841	Oxygen-evolving enhancer protein 1-2	0.96	0.88
PGSC0003DMP400029964	Heterogeneous nuclear ribonucleoprotein 27C	0.61	0.79
P50433	Serine hydroxymethyltransferase	0.62	0.74
P93566	Oxygen-evolving enhancer protein 2	0.85	0.73
PGSC0003DMP400021624	PRPL11	0.87	0.72
Q00321	Chlorophyll a-b binding protein	0.83	0.72
PGSC0003DMP400046718	NAD dependent epimerase	0.75	0.58
Q2MI64	50S ribosomal protein L14	0.82	0.58
Q38HV4	Fructose-bisphosphate aldolase	1.91	NA
PGSC0003DMP400038572	Fructose-bisphosphate aldolase	1.69	NA
PGSC0003DMP400003396	Non-specific lipid-transfer protein 1	1.57	NA
C9EFD1	Chloroplast ribosomal protein	1.24	NA
A0MWB6	Transcriptional coactivator multiprotein bridging factor	1.09	NA
PGSC0003DMP400041249	EMB2394	0.95	NA
PGSC0003DMP400025698	High mobility group protein	0.87	NA
P26320	Oxygen-evolving enhancer protein 1	0.78	NA
PGSC0003DMP400007506	Photosystem Q	0.7	NA
C5MR70	Chloroplast manganese stabilizing protein-II	0.45	NA
B0ZTE3	Starch synthase	NA	4.41
PGSC0003DMP400037406	21kD protein	NA	1.90
PGSC0003DMP400015440	Ferritin	NA	1.90
Q6RFS8	Catalase	NA	1.42
PGSC0003DMP400048120	Photosystem I subunit XI	NA	1.38
Q5NE20	Carbonic anhydrase	NA	1.36
P26575	Rubisco small chain 2A	NA	1.31
Q308A9	Ferritin	NA	1.28
PGSC0003DMP400015694	Heat shock protein 70	NA	1.20
Q8RXK6	DEAD-box RNA helicase 8	NA	1.11
PGSC0003DMP400006368	Ferredoxin--NADP reductase	NA	1.10
Q2PYW5	Catalase	NA	0.99
PGSC0003DMP400025425	Gene of unknown function	NA	0.75

**Table 4:** Proteins from two ETI interactions with decreased abundance (ETI-IpiO and ETI-Avr2), 18 hpi (hours post infiltration). A comparison is made between immune-treated plants and the control plants infiltrated with medium only. All values are in log2 fold. Non-significant *p* values are indicated by NA. All *p*-values < 0.01 are shown in the table.

Protein ID	Protein Name	Degree of Regulation (log2)	
		ETI (Blb1-IpiO)	ETI (AVR2-R2)
E1AXT5	Apoplastic invertase	−0.40	−0.49
P49316	Catalase isozyme 2	−0.66	−0.63
PGSC0003DMP400032195	SWIb domain-containing protein	−0.46	−0.63
PGSC0003DMP400000868	Actin-11	−0.70	−0.65
O49074	Ribulose bisphosphate carboxylase	−0.64	−0.66
Q5MA02	Cytochrome c oxidase subunit 2	−0.91	−0.66
PGSC0003DMP400000788	Inner membrane protein PPF-1	−0.62	−0.73
PGSC0003DMP400010545	O-glycosyl hydrolase	−0.56	−0.74
PGSC0003DMP400046981	Kunitz trypsin inhibitor	−0.94	−0.75
PGSC0003DMP400025093	ATP-dependent Clp protease	−0.66	−0.75
PGSC0003DMP400039372	AGO4-2	−0.63	−0.82
Q42871	Endoglucanase	−1.64	−0.82
PGSC0003DMP400030598	Ribonucleoprotein	−0.96	−0.86
A0A024J2E4	Putative transcription activator TraR	−0.90	−1.22
PGSC0003DMP400040149	Short chain alcohol dehydrogenase	−1.23	−0.92
PGSC0003DMP400019158	Aspartate aminotransferase	−0.79	−0.93
PGSC0003DMP400017124	Ferredoxin-dependent glutamate synthase 1	−1.24	−0.95
PGSC0003DMP400018521	Subtilase	−0.97	−1.04
PGSC0003DMP400042601	Gene of unknown function	−0.66	−1.04
D0EJY9	Molecular chaperone Hsp90-3	−1.29	−1.08
P32811	Alpha-glucan phosphorylase	−1.47	−1.10
Q1EBW2	Aspartate aminotransferase	−0.91	−1.13
PGSC0003DMP400018523	Subtilase	−0.84	−1.13
PGSC0003DMP400015799	Basic 7S globulin 2 small subunit	−0.98	−1.15
A7LKN1	TAO1	−1.12	−1.16
F4HRC1	THO complex subunit 5A	−0.87	−1.18
PGSC0003DMP400068875	Threonyl-tRNA synthetase	−1.21	−1.19
PGSC0003DMP400039983	Protein SIS1	−1.81	−1.20
PGSC0003DMP400014905	Polygalacturonase inhibiting protein	−0.88	−1.24
PGSC0003DMP400011487	GTP-binding nuclear protein Ran1	−1.15	−1.29
PGSC0003DMP400007007	P69B protein	−1.80	−1.42
PGSC0003DMP400033260	Xylem serine proteinase 1	−2.19	−1.59
PGSC0003DMP400040582	Biotin carboxylase carrier protein	−1.18	−1.61
PGSC0003DMP400032609	Amidase family protein	−0.50	NA
PGSC0003DMP400044937	Serine carboxypeptidase	−0.74	NA
PGSC0003DMP400034518	Chitinase	−0.77	NA
PGSC0003DMP400013944	Eukaryotic translation initiation factor 4F	−0.88	NA
PGSC0003DMP400046710	Lysyl-tRNA synthetase	−0.90	NA
PGSC0003DMP400002882	Glycine-rich protein 2	−0.90	NA
PGSC0003DMP400001015	Class III peroxidase	−0.93	NA
PGSC0003DMP400012991	Apyrase 3	−0.93	NA
PGSC0003DMP400043401	NADPH:protochlorophyllide oxidoreductase	−1.00	NA
F4IFG1	Dynamin related protein	−1.02	NA
PGSC0003DMP400056894	P69B protein	−1.32	NA
PGSC0003DMP400012143	Gene of unknown function	−1.39	NA
PGSC0003DMP400009992	Beta tubulin	−1.43	NA
PGSC0003DMP400036604	WPP domain-associated protein	−1.45	NA
PGSC0003DMP400033261	Xylem serine proteinase 1	−1.55	NA
PGSC0003DMP400032897	Aspartic proteinase nepenthesin-1	−2.48	NA
B9JM45	6-Phosphogluconate dehydrogenase	NA	−1.31
PGSC0003DMP400020641	CBL-interacting protein kinase 13	NA	0.71
Q8W174	Peroxidase	NA	0.70
PGSC0003DMP400016778	Periplasmic beta-glucosidase	NA	0.62
Q2MI54	30S ribosomal protein S7	NA	0.62
PGSC0003DMP400045032	Pectinesterase	NA	0.60
E2I6L5	Polyubiquitin	NA	0.59
PGSC0003DMP400023756	GDSL-lipase protein	NA	0.58
PGSC0003DMP400009676	Eukaryotic translation initiation factor 2 family protein	NA	0.58
PGSC0003DMP400045566	50S ribosomal protein L19-2	NA	0.58
PGSC0003DMP400064549	Subtilisin-like protease	NA	0.55
Q3LS00	Polygalacturonase inhibitor	NA	0.54
C0Z2Q9	AT3G13920 protein	NA	0.53
PGSC0003DMP400014290	AMP dependent CoA ligase	NA	0.53
PGSC0003DMP400009317	Superoxide dismutase	NA	0.53
F4JWP8	Homeobox protein knotted-1-like 3	NA	0.53
Q9LXG7	Aldose 1-epimerase family protein	NA	0.52
PGSC0003DMP400006170	60S ribosomal protein L7A	NA	0.51
Q43286	Histone H2A	NA	0.50
PGSC0003DMP400042811	60S ribosomal protein L18	NA	0.41
PGSC0003DMP400002092	Histone H1F	NA	0.37
PGSC0003DMP400011213	Beta-glucosidase 01	NA	0.32
PGSC0003DMP400042879	Histone H2A.1	NA	0.26
PGSC0003DMP400034568	Histone H1	NA	0.07

**Table 5:** Examples of methylated peptides identified in the analysis (all methylated peptides are shown in the supplementary material 1. Regulation in immunity: whether the protein was identified as regulated in the quantitative analysis. Sequence: The sequence of the modified peptide, underlined is the methylated site. The position and nature of the modified amino acid are indicated by a number. In the cases where a methylated peptide has been identified but the site not determined, this is indicated by NA.

External Id	Protein Name	Regulation in Immunity	Sequence	Modification
P50433	Serine hydroxymethyltransferase	Up in ETI	YSEGYPGAR	Dimethyl(R)
P25079	Ribulose bisphosphate carboxylase large chain	Up in ETI	DTDILAAFR	Dimethyl(R)
			DITLGFVDLLR	Dimethyl(R)
Q2VEH0	ATP synthase subunit beta	Up in PTI	MRINPTTSGSGVSTLEK	Methyl(R), Oxidation(M)
			FLSQPFFVAEVFTGSPGKYVGLAETIR	Dimethyl(R)
			FLSQPFFVAEVFTGSPGKYVGLAETIR	Methyl(K), Methyl(R)
Q9LEB0	Pectinesterase	Down in PTI	SNTIITGSR	Methyl(R)
PGSC0003DMP400046973	UPF0497 membrane protein	Up in PTI and ETI	YVNGFVDTIETTGIDTFEELR	Dimethyl(R)
PGSC0003DMP400002077	Histone H3.2	NA	FQSSAVAALQEAAEAYLVGVFEDTNLCAIHAK	Methyl(K)

## Supplementary Materials

Supplementary materials can be found at https://www.mdpi.com/1422-0067/20/19/4726/s1.

## Abbreviations

PAMP	Pathogen-associated molecular pattern
PRR	Pattern-recognition receptors
PTI	PAMP-triggered immunity
ROS	Reactive oxygen species
ETS	Effector-triggered susceptibility
ETI	Effector-triggered immunity
HR	Hypersensitive response

## References

[1] Zipfel C. (2008). Pattern-recognition receptors in plant innate immunity. Curr. Opin. Immunol..

[2] Baxter A., Mittler R., Suzuki N. (2013). ROS as key players in plant stress signalling. J. Exp. Bot..

[3] Pitzschke A., Schikora A., Hirt H. (2009). MAPK cascade signalling networks in plant defence. Curr. Opin. Plant Biol..

[4] Jones J.D.G., Dangl J.L. (2006). The plant immune system. Nature.

[5] Coll N.S., Epple P., Dangl J.L. (2011). Programmed cell death in the plant immune system. Cell Death Differ..

[6] Thomma B.P.H.J., Nürnberger T., Joosten M.H.A.J. (2011). Of PAMPs and Effectors: The Blurred PTI-ETI Dichotomy. Plant Cell.

[7] Leibman-Markus M., Pizarro L., Schuster S., Lin Z.J.D., Gershony O., Bar M., Coaker G., Avni A. (2018). The intracellular nucleotide-binding leucine-rich repeat receptor (SlNRC4a) enhances immune signalling elicited by extracellular perception. Plant Cell Environ..

[8] Lee S., Fu F., Xu S., Lee S.Y., Yun D.-J., Mengiste T. (2016). Global Regulation of Plant Immunity by Histone Lysine Methyl Transferases. Plant Cell.

[9] Haverkort A.J., Struik P.C., Visser R.G.F., Jacobsen E. (2009). Applied Biotechnology to Combat Late Blight in Potato Caused by Phytophthora Infestans. Potato Res..

[10] Champouret N., Bouwmeester K., Rietman H., van der Lee T., Maliepaard C., Heupink A., van de Vondervoort P.J.I., Jacobsen E., Visser R.G.F., van der Vossen E.A.G. (2009). Phytophthora infestans Isolates Lacking Class I ipiO Variants Are Virulent on Rpi-blb1 Potato. Mol. Plant-Microbe Interact..

[11] Lokossou A.A., Park T.-h., van Arkel G., Arens M., Ruyter-Spira C., Morales J., Whisson S.C., Birch P.R.J., Visser R.G.F., Jacobsen E. (2009). Exploiting Knowledge of R/Avr Genes to Rapidly Clone a New LZ-NBS-LRR Family of Late Blight Resistance Genes from Potato Linkage Group IV. Mol. Plant-Microbe Interact..

[12] Vleeshouwers V.G.A.A., Rietman H., Krenek P., Champouret N., Young C., Oh S.-K., Wang M., Bouwmeester K., Vosman B., Visser R.G.F. (2008). Effector Genomics Accelerates Discovery and Functional Profiling of Potato Disease Resistance and Phytophthora Infestans Avirulence Genes. PLoS ONE.

[13] Turnbull D., Yang L., Naqvi S., Breen S., Welsh L., Stephens J., Morris J., Boevink P.C., Hedley P.E., Zhan J. (2017). RXLR Effector AVR2 Up-Regulates a Brassinosteroid-Responsive bHLH Transcription Factor to Suppress Immunity. Plant Physiol..

[14] Saunders D.G.O., Breen S., Win J., Schornack S., Hein I., Bozkurt T.O., Champouret N., Vleeshouwers V.G.A.A., Birch P.R.J., Gilroy E.M. (2012). Host Protein BSL1 Associates with *Phytophthora infestans* RXLR Effector AVR2 and the *Solanum demissum* Immune Receptor R2 to Mediate Disease Resistance. Plant Cell.

[15] Burra D.D., Lenman M., Levander F., Resjö S., Andreasson E. (2018). Comparative Membrane-Associated Proteomics of Three Different Immune Reactions in Potato. Int. J. Mol. Sci..

[16] Van Aubel G., Buonatesta R., Van Cutsem P. (2013). Cos-oga, a new oligosaccharidic elicitor that induces protection against a wide range of plant pathogens. IOBC-WPRS Bull..

[17] Rayapuram C., Jensen M.K., Maiser F., Shanir J.V., Hornshøj H., Rung J.H., Gregersen P.L., Schweizer P., Collinge D.B., Lyngkjær M.F. (2012). Regulation of basal resistance by a powdery mildew-induced cysteine-rich receptor-like protein kinase in barley. Mol. Plant Pathol..

[18] Xiao C., Gao J., Zhang Y., Wang Z., Zhang D., Chen Q., Ye X., Xu Y., Yang G., Yan L. (2019). Quantitative Proteomics of Potato Leaves Infected with Phytophthora infestans Provides Insights into Coordinated and Altered Protein Expression during Early and Late Disease Stages. Int. J. Mol. Sci..

[19] Afitlhile M.M., Fukushige H., Nishimura M., Hildebrand D.F. (2005). A defect in glyoxysomal fatty acid β-oxidation reduces jasmonic acid accumulation in Arabidopsis. Plant Physiol. Biochem..

[20] Davidson R.M., Reeves P.A., Manosalva P.M., Leach J.E. (2009). Germins: A diverse protein family important for crop improvement. Plant Sci..

[21] Lane B.G. (2002). Oxalate, Germins, and Higher-Plant Pathogens. IUBMB Life.

[22] Grosse-Holz F., Kelly S., Blaskowski S., Kaschani F., Kaiser M., van der Hoorn R.A.L. (2018). The transcriptome, extracellular proteome and active secretome of agroinfiltrated Nicotiana benthamiana uncover a large, diverse protease repertoire. Plant Biotechnol. J..

[23] Roppolo D., De Rybel B., Tendon V.D., Pfister A., Alassimone J., Vermeer J.E.M., Yamazaki M., Stierhof Y.-D., Beeckman T., Geldner N. (2011). A novel protein family mediates Casparian strip formation in the endodermis. Nature.

[24] Serrazina S., Santos C., Machado H., Pesquita C., Vicentini R., Pais M.S., Sebastiana M., Costa R. (2015). Castanea root transcriptome in response to Phytophthora cinnamomi challenge. Tree Genet. Genomes.

[25] Nes W.D. (1987). Biosynthesis and Requirement for Sterols in the Growth and Reproduction of Oomycetes. Ecology and Metabolism of Plant Lipids.

[26] Thomas E.L., Van der Hoorn R.A.L. (2018). Ten Prominent Host Proteases in Plant-Pathogen Interactions. Int. J. Mol. Sci..

[27] Greer E.L., Shi Y. (2012). Histone methylation: A dynamic mark in health, disease and inheritance. Nat. Rev. Genet..

[28] Ng A., Xavier R.J. (2011). Leucine-rich repeat (LRR) proteins: Integrators of pattern recognition and signaling in immunity. Autophagy.

[29] Padmanabhan M., Cournoyer P., Dinesh-Kumar S.P. (2009). The leucine-rich repeat domain in plant innate immunity: A wealth of possibilities. Cell. Microbiol..

[30] Bengtsson T., Weighill D., Proux-Wéra E., Levander F., Resjö S., Burra D.D., Moushib L.I., Hedley P.E., Liljeroth E., Jacobson D. (2014). Proteomics and transcriptomics of the BABA-induced resistance response in potato using a novel functional annotation approach. BMC Genom..

[31] Lionetti V., Cervone F., Bellincampi D. (2012). Methyl esterification of pectin plays a role during plant–pathogen interactions and affects plant resistance to diseases. J. Plant Physiol..

[32] Marty P., Jouan B., Bertheau Y., Vian B., Goldberg R. (1997). Charge density in stem cell walls of Solanum tuberosum genotypes and susceptibility to blackleg. Phytochemistry.

[33] McMillan G.P., Hedley D., Fyffe L., Pérombelon M.C.M. (1993). Potato resistance to soft-rot erwinias is related to cell wall pectin esterification. Physiol. Mol. Plant Pathol..

[34] Li J., Brader G., Palva E.T. (2008). Kunitz Trypsin Inhibitor: An Antagonist of Cell Death Triggered by Phytopathogens and Fumonisin B1 in Arabidopsis. Mol. Plant.

[35] Lam E., Kato N., Lawton M. (2001). Programmed cell death, mitochondria and the plant hypersensitive response. Nature.

[36] Miller G., Shulaev V., Mittler R. (2008). Reactive oxygen signaling and abiotic stress. Physiol. Plant..

[37] Sharma P., Jha A.B., Dubey R.S., Pessarakli M. (2012). Reactive Oxygen Species, Oxidative Damage, and Antioxidative Defense Mechanism in Plants under Stressful Conditions. J. Bot..

[38] Du Y.-Y., Wang P.-C., Chen J., Song C.-P. (2008). Comprehensive Functional Analysis of the Catalase Gene Family in Arabidopsis thaliana. J. Integr. Plant Biol..

[39] Sun T., Liu F., Wang W., Wang L., Wang Z., Li J., Que Y., Xu L., Su Y. (2018). The Role of Sugarcane Catalase Gene ScCAT2 in the Defense Response to Pathogen Challenge and Adversity Stress. Int. J. Mol. Sci..

[40] Staiger D., Korneli C., Lummer M., Navarro L. (2013). Emerging role for RNA-based regulation in plant immunity. New Phytol..

[41] Li D., Zhang H., Liu H., Wang X., Song F. (2008). OsBIRH1, a DEAD-box RNA helicase with functions in modulating defence responses against pathogen infection and oxidative stress. J. Exp. Bot..

[42] Restrepo S., Myers K.L., del Pozo O., Martin G.B., Hart A.L., Buell C.R., Fry W.E., Smart C.D. (2005). Gene Profiling of a Compatible Interaction Between Phytophthora infestans and Solanum tuberosum Suggests a Role for Carbonic Anhydrase. Mol. Plant-Microbe Interact..

[43] Lukasik E., Takken F.L.W. (2009). STANDing strong, resistance proteins instigators of plant defence. Curr. Opin. Plant Biol..

[44] Real M.D., Company P., García-Agustín P., Bennett A.B., González-Bosch C. (2004). Characterization of tomato endo-β-1,4-glucanase Cel1 protein in fruit during ripening and after fungal infection. Planta.

[45] Flors V., Leyva M.d.l.O., Vicedo B., Finiti I., Real M.D., García-Agustín P., Bennett A.B., González-Bosch C. (2007). Absence of the endo-β-1,4-glucanases Cel1 and Cel2 reduces susceptibility to Botrytis cinerea in tomato. Plant J..

[46] ZEIER J. (2013). New insights into the regulation of plant immunity by amino acid metabolic pathways. Plant Cell Environ..

[47] Serrano I., Buscaill P., Audran C., Pouzet C., Jauneau A., Rivas S. (2016). A non canonical subtilase attenuates the transcriptional activation of defence responses in Arabidopsis thaliana. eLife.

[48] Mahadevan C., Krishnan A., Saraswathy G.G., Surendran A., Jaleel A., Sakuntala M. (2016). Transcriptome- Assisted Label-Free Quantitative Proteomics Analysis Reveals Novel Insights into Piper nigrum—Phytophthora capsici Phytopathosystem. Front. Plant Sci..

[49] Alban C., Tardif M., Mininno M., Brugière S., Gilgen A., Ma S., Mazzoleni M., Gigarel O., Martin-Laffon J., Ferro M. (2014). Uncovering the Protein Lysine and Arginine Methylation Network in Arabidopsis Chloroplasts. PLoS ONE.

[50] Issakidis-Bourguet E., Shen Y., Zhou D.-X. (2016). Perspectives on the interactions between metabolism, redox, and epigenetics in plants. J. Exp. Bot..

[51] Locasale J.W. (2013). Serine, glycine and one-carbon units: Cancer metabolism in full circle. Nat. Rev. Cancer.

[52] Zhang H., Deng X., Miki D., Cutler S., La H., Hou Y.-J., Oh J., Zhu J.-K. (2012). Sulfamethazine Suppresses Epigenetic Silencing in *Arabidopsis* by Impairing Folate Synthesis. Plant Cell.

[53] Rojas C., Senthil-Kumar M., Tzin V., Mysore K. (2014). Regulation of primary plant metabolism during plant-pathogen interactions and its contribution to plant defense. Front. Plant Sci..

[54] Abreha K.B., Alexandersson E., Vossen J.H., Anderson P., Andreasson E. (2015). Inoculation of Transgenic Resistant Potato by Phytophthora infestans Affects Host Plant Choice of a Generalist Moth. PLoS ONE.

[55] Lenman M., Ali A., Mühlenbock P., Carlson-Nilsson U., Liljeroth E., Champouret N., Vleeshouwers V.G.A.A., Andreasson E. (2016). Effector-driven marker development and cloning of resistance genes against Phytophthora infestans in potato breeding clone SW93-1015. Theor. Appl. Genet..

[56] Van Der Vossen E., Sikkema A., Hekkert B.t.L., Gros J., Stevens P., Muskens M., Wouters D., Pereira A., Stiekema W., Allefs S. (2003). An ancient R gene from the wild potato species Solanum bulbocastanum confers broad-spectrum resistance to Phytophthora infestans in cultivated potato and tomato. Plant J..

[57] Du J., Rietman H., Vleeshouwers V.G.A.A. (2014). Agroinfiltration and PVX agroinfection in potato and Nicotiana benthamiana. J. Vis. Exp. JoVE.

[58] Kessner D., Agus D., Chambers M., Mallick P., Burke R. (2008). ProteoWizard: Open source software for rapid proteomics tools development. Bioinformatics.

[59] The Potato Genome Sequencing C., Xu X., Pan S., Cheng S., Zhang B., Mu D., Ni P., Zhang G., Yang S., Li R. (2011). Genome sequence and analysis of the tuber crop potato. Nature.

[60] Elias J.E., Gygi S.P. (2007). Target-decoy search strategy for increased confidence in large-scale protein identifications by mass spectrometry. Nat. Methods.

[61] Häkkinen J., Vincic G., Månsson O., Wårell K., Levander F. (2009). The Proteios Software Environment: An Extensible Multiuser Platform for Management and Analysis of Proteomics Data. J. Proteome Res..

[62] Käll L., Storey J.D., MacCoss M.J., Noble W.S. (2008). Assigning Significance to Peptides Identified by Tandem Mass Spectrometry Using Decoy Databases. J. Proteome Res..

[63] Sandin M., Krogh M., Hansson K., Levander F. (2011). Generic workflow for quality assessment of quantitative label-free LC-MS analysis. Proteomics.

[64] Teleman J., Chawade A., Sandin M., Levander F., Malmström J. (2016). Dinosaur: A Refined Open-Source Peptide MS Feature Detector. J. Proteome Res..

[65] Sandin M., Ali A., Hansson K., Månsson O., Andreasson E., Resjö S., Levander F. (2013). An Adaptive Alignment Algorithm for Quality-controlled Label-free LC-MS. Mol. Cell. Proteom..

[66] Smyth G.K. (2005). Limma: Linear Models for Microarray Data. Bioinform. Comput. Biol. Solut. Using R Bioconductor.

[67] Chawade A., Alexandersson E., Levander F. (2014). Normalyzer: A Tool for Rapid Evaluation of Normalization Methods for Omics Data Sets. J. Proteome Res..

[68] Taverner T., Karpievitch Y.V., Polpitiya A.D., Brown J.N., Dabney A.R., Anderson G.A., Smith R.D. (2012). DanteR: An extensible R-based tool for quantitative analysis of -omics data. Bioinformatics.

[69] Savitski M.M., Lemeer S., Boesche M., Lang M., Mathieson T., Bantscheff M., Kuster B. (2011). Confident Phosphorylation Site Localization Using the Mascot Delta Score. Mol. Amp. Cell. Proteom..

[70] Pfaffl M.W. (2001). A new mathematical model for relative quantification in real-time RT-PCR. Nucleic Acids Res..

[71] Rozen S., Skaletsky H. (2000). Primer3 on the WWW for General Users and for Biologist Programmers. Bioinform. Methods Protoc..

[72] Chawade A., Alexandersson E., Bengtsson T., Andreasson E., Levander F. (2016). Targeted Proteomics Approach for Precision Plant Breeding. J. Proteome Res..

[73] Ghislain M., Byarugaba A.A., Magembe E., Njoroge A., Rivera C., Román M.L., Tovar J.C., Gamboa S., Forbes G.A., Kreuze J.F. (2019). Stacking three late blight resistance genes from wild species directly into African highland potato varieties confers complete field resistance to local blight races. Plant Biotechnol. J..

