# Supplementary material for: Proteomics of PTI and Two ETI Immune Reactions in Potato Leaves
Source: Int J Mol Sci. 2019 Sep 24;20(19):4726. doi: 10.3390/ijms20194726 (PMC6802228; doi:10.3390/ijms20194726)
Supplement: Supplementary file 1 [file ijms-20-04726-s001.zip › ijms-554174-SI/supple 1,2/ijms-554174-supplementary 2 proofed.docx]

Supplementary material 2

Proteomics of PTI and Two ETI Immune Reactions in Potato Leaves

Svante Resjö, Muhammad Awais Zahid, Dharani Dhar Burra, Marit Lenman, Fredrik Levander and Erik Andreasson

**Table S1.** Spectrophotometric values of RNA samples for quality check. RNA samples were measured on Nano Drop™ 2000/2000c (Thermofisher Waltham, MA, USA).

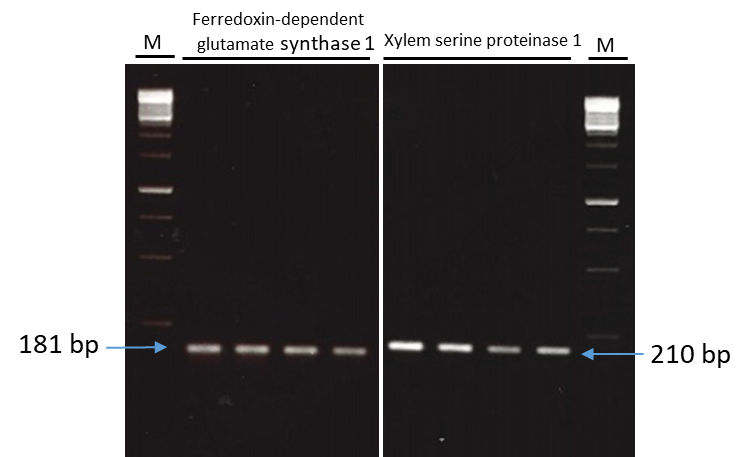


**Figure S1.** Agarose gel electrophoresis of qPCR products show single amplicons. Analysis of qPCR amplicons on an agarose gel shows a single band for both Ferredoxin dependent glutamate synthase 1 gene (181bp) and Xylem serine proteinase 1 gene (210bp).

**
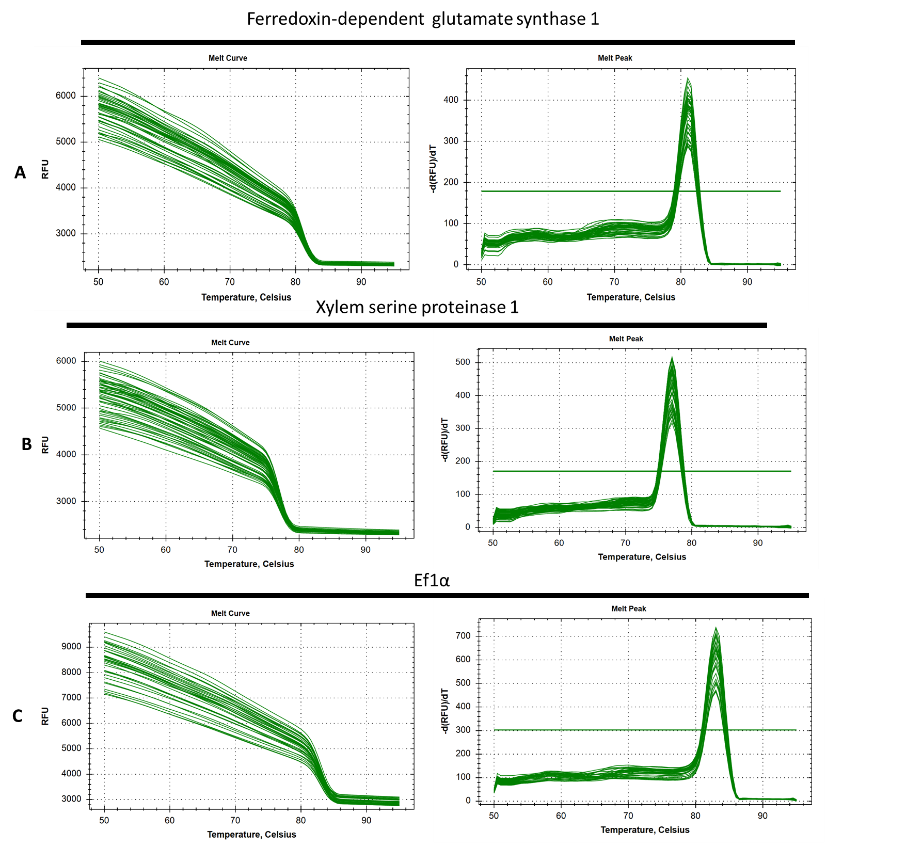

Figure S2.** Melting curves from qPCR. Bio-Rad CFX Manager™ Software was used to visualize melt and peak curves. It reveals a single peak following melt curve analysis. (**A**) Primer efficiency for Ferredoxin-dependent glutamate synthase 1 gene was 100.80 %, primer efficiency was calculated according to the pfaffl method. (**B**) Primer efficiency for Xylem serine proteinase 1 was 95.30 %. (**C**) Primer efficiency for Ef1α was 91.29 %.
